# Physical performance and 25-hydroxyvitamin D: a cross-sectional study of pregnant Swedish and Somali immigrant women and new mothers

**DOI:** 10.1186/1471-2393-13-237

**Published:** 2013-12-17

**Authors:** Paul Kalliokoski, Yngve Bergqvist, Monica Löfvander

**Affiliations:** 1 Primary Care Center Jakobsgårdarna, Jaxtorget 7A, Box 100 33 Borlänge S-781 10, Sweden; 2 Center for Clinical Research Dalarna, Falun, Sweden; 3 Center for Clinical Research Västmanland, Uppsala University, Västerås, Sweden; 4 Family Medicine and Preventive Medicine Unit, Department of Public Health and Caring Sciences, Uppsala University, Uppsala, Sweden; 5 Center of Family Medicine, Department of Neurobiology, Care Sciences and Society, Karolinska Institutet, Huddinge, Sweden

**Keywords:** Vitamin D, 25(OH)D, Ultraviolet B radiation, Grip strength, Clinical observation, Physical performance, Primary care, Somalia, Pregnancy

## Abstract

**Background:**

Severe vitamin D deficiency can impair muscle strength. The study aims were to examine physical performance in the hands and upper legs, and analyze plasma 25-hydroxyvitamin D (25(OH)D) concentrations in women with presumably low (veiled, Somali-born) and high levels (unveiled, Swedish-born).

**Methods:**

Women (n=123, 58% Swedish) enrolled at a Swedish antenatal clinic, latitude 60° N, were recruited. Plasma 25(OH) D was analyzed, measured as nmol/L, then categorized as <10 = undetectable, 10–24, 25–49, 50–74 or >75. Muscle strength was tested: maximal hand grip strength (in Newtons, N), and upper leg performance (categorized as able/unable to perform squatting, standing on one leg, standing from a chair, and lifting their hips). Social and anthropometric data were collected. Non-parametric statistics tested the data for differences in their ability to perform the tests across 25(OH)D categories. Undetectable values (<10 nmol/L) were replaced with ‘9’ in the linear correlation statistics. A final main effect model for grip strength (in N) was calculated using stepwise linear regression for independent variables: country of birth, 25(OH)D levels, age, height, weight, physical activity, lactation status, parity, and gestational age.

**Results:**

Somali participants (35%) had 25(OH)D levels of <10 nmol/L, and 90% had <25 nmol/L; 10% of Swedish participants had <25 nmol/L of 25(OH)D, and 54% had <50 nmol/L. Somali women had a relatively weak grip strength compared with Swedish women: median 202 N (inter-quartile range 167–246) vs. median 316 N (inter-quartile range 278–359), respectively. Somali women were also weak in upper leg performance: 73% were unable to squat, 29% unable to stand on one leg, and 21% could not lift their hips (not significant across 25(OH)D categories); most Swedish women could perform these tests. In the final model, grip strength (N) was significantly associated with 25(OH)D levels (B 0.94, p=0.013) together with Somali birth (B −63.9, p<0.001), age (B 2.5, p=0.02) and height (B 2.6, p=0.01).

**Conclusions:**

Many Somali women had undetectable/severely low 25(OH)D concentrations and pronounced hand and upper leg weakness; grip strength was strongly associated with 25(OH)D. Maternity health care personnel should be aware of this increased frequency and manage care accordingly.

## Background

Ultraviolet B radiation induces skin synthesis of vitamin D3. The sun is the most important source of ultraviolet B radiation. Preventing this radiation from reaching the skin reduces vitamin D3 production [1,2]. The heart of Scandinavia lies on the 60^th^ parallel, where the angle of the sun limits vitamin D3 production to six months around summer [3]; for the remaining part of the year the ultraviolet B radiation is absorbed by the atmosphere. Darkly-pigmented persons who wear veiled clothing and who live at higher latitudes often produce insufficient vitamin D3 during much of the year [2]. Foods such as oily fish, milk, egg yolk, and some mushrooms, are a secondary source of vitamin D3 [4].

Ultraviolet B radiation transforms 7-dehydrocholesterol to vitamin D3 in the skin. Vitamin D3 is hydroxylated to 25-hydroxyvitamin D (25(OH)D) in the liver and then transported by a vitamin D binding globulin to muscle cells where it is activated to calcitriol [2,5,6]. 25(OH)D has an influence on enzyme systems within muscle cells [5,7,8]; 25(OH)D deficiency may lead to symptoms of myopathy [9,10]. Polymorphisms in the vitamin D receptor account for individual differences in skeletal muscle strength [11]. The anabolic effects of 25(OH)D are related to increased muscle volume and enhanced contraction and relaxation of muscles [2], while 25(OH)D deficiency may cause atrophy and weakness [12,13]. Vitamin D3 supplements may improve muscle strength when given to elderly persons [14], and to younger people who are 25(OH)D deficient (<25 nmol/L) [14].

Skin synthesis of vitamin D3 decreases with age [8]. As a consequence, many studies have tried to evaluate the vitamin D status of elderly persons and their lower extremity function [7,15-20]. Little is known about the hand strength and/or physical performance in pregnant women, or their 25(OH)D status, possibly because pregnant and lactating women are often excluded from studies [21]. Despite this, gestational diabetes, pre-eclampsia [22] and birth complications have been tentatively linked to vitamin D insufficiency in US studies [23-29].

A historic case report reported on osteomalacia patients with a waddling gait and muscle weakness [30]. The first author of this report has also observed a waddling gait among his female patients born outside of Sweden, especially women coming from Somalia (unpublished data). In Sweden, there are few data regarding vitamin D status in the general population and pregnant women, and no information about the possible consequences of vitamin D deficiency. One recent Swedish study showed that pregnant Somali women often have severe vitamin D deficiency, but no clinical data were presented [31]. Severe vitamin D deficiency may also impair muscle strength in younger women and interfere with activities of daily living, for example childcare. Immigrant children have a high frequency of unintentional injuries according to Scandinavian studies [32-34]. These range of factors contributed to the rationale for a project to explore physical performance and 25(OH)D levels in pregnant women, with the focus on immigrant women, and darkly-pigmented and veiled women in particular. The overall aim of the project was to improve the function and lifestyle of all pregnant women and new mothers by providing them with adequate antenatal and primary health care. The primary outcome variables were grip strength, physical performance of the lower limbs, and plasma concentrations of 25(OH)D in a group of pregnant women and new mothers with presumably low 25(OH)D concentrations (i.e. veiled, Somali-born women) and high concentrations (i.e. unveiled, Swedish-born women).

We hypothesized that grip strength and physical performance in the upper legs would correlate with 25(OH)D levels, and that this correlation would be more evident in the lower 25(OH)D concentration ranges, which are often measured in Somali women.

## Subjects and methods

### Recruitment procedure

This study was designed as a cross-sectional study. It was conducted in a primary care antenatal clinic located in a mid-sized Swedish industrial town on the 60^th^ parallel.

We chose a study period in the late spring to reflect the effects of the dark season, when low levels of ultraviolet B radiation affects 25(OH)D production, especially in darkly-pigmented or veiled persons. A retrospective design was chosen to minimize the seasonal effects of ultraviolet B radiation. This design required women at a specific time of the year, rather than women at a specific gestational age; this enabled us to complete the blood sampling and physical tests during a limited period of time. In doing so, the study population included women in all trimesters, as well as new mothers.

Sixty participants were needed (30 Swedish; 30 Somali) to reach 94% power and α error of 0.05, according to a power calculation using data from an unpublished pilot study. This study of 25(OH)D in consecutive female patients (n =30 veiled; n = 12 unveiled) had mean 25(OH)D values of 17 nmol/L (Standard deviation 13.0) and 35 nmol/L (Standard deviation 28.0), respectively. The power calculation was approved by the regional ethics committee at Uppsala (D nr 2010/40), Sweden.

The study group was recruited retrospectively from Somali and Swedish women enrolled at an antenatal clinic from May 2010 over 21 months (Somali) and 9 months (Swedish). A retrospective enlisting procedure was performed to reach statistical power. Therefore, the initial study population that was needed equated to roughly 75 Swedish and at least 50 Somali women.

Women <18 years old were excluded, as were women from countries other than Sweden and Somalia and those who had severe mental or somatic disorders. Clothing that covered the arms, legs and head was considered to be concealing (i.e. veiled clothing).

### Overview of sampling procedure

A list was provided by the antenatal clinic containing the names and parity of 118 Somali women (from the previous 21 months) and 309 Swedish women (from the previous 9 months). The country of birth was identified by a Somali assistant nurse and the head researcher, using the criterion of surname and language. All the women were categorized as primi- or multi-para. The Swedish women were then randomized to match the Somali women by categorized parity.

All of the participants were sent an information letter in Swedish. In addition, a Somali version of the letter was sent to the Somali women. The Somali women were contacted by phone by a Somali nurse assistant, who read the information in Somali if the woman had low literacy; the blood sampling dates were provided and written consent was offered to the women who wanted to participate. The Swedish women were contacted by one of the research doctors via phone.

A total of 140 women (out of 217 that were contacted, or 64.5%) presented for the blood sampling and consenting (82 Swedish; 58 Somali). Participants were given a scheduled time for ten days later, with one of two research doctors, for physical tests, questionnaires and information. This time interval was required for the distribution and analyses of blood samples.

Finally, a total of 123 women (56.7% or 123/217) returned to the doctors where they completed the questionnaires and physical tests; these women constituted the final study population (71 Swedish; 52 Somali women). There were no statistical differences between the participating women and the non-participants.

## Methods

Venous blood samples were collected and centrifuged. Serum vitamin D was measured using Lisason 25 OH Vitamin D total assay (DiaSorin, Stillwater, Minnesota, USA) at the Clinical Chemistry Laboratory at the University Hospital, Uppsala, Sweden, a certified laboratory. The other assays (ALP, PTH, Ca, albumin) were measured using Abbott Architect ci8200 (Abbott Laboratories, Green Oaks, Illinois, USA) at the Department of Clinical Chemistry, Falun Hospital, Sweden. Hemoglobin and glucose were measured by HemoCue System (HemoCue Sweden, Ängelholm, Sweden) at an antenatal clinic that was quality accredited. The alkaline phosfates (ALP), parathyroid hormone (PTH) and free serum calcium were analyzed to monitor metabolic skeletal activity. Hemoglobin and glucose were measured in the baseline blood samples in order to screen for non-vitamin-D-related reasons for fatigue and muscular weakness.

Anthropometric measurements were performed and questionnaires were administered by the doctors about the participant’s lifestyle, pain and medication history, concurrent diseases, socio-cultural variables, caesarean section history, gestational age, and lactation history. Physical activity was self-reported and measured as 0, 1, 2 (seldom) or ≥3 times weekly (often). The participants then performed a range of physical tests.

General advice about adequate sun exposure and food was given by the doctors towards the end of the encounter. Finally, the doctor opened a sealed envelope containing the results of the 25(OH)D tests, and the women with 25(OH)D levels <50 nmol/L were prescribed standard preparations of vitamin D and calcium. Thus, the doctors were blinded until this point in relation to the blood test results.

### Grip strength and physical performance

Tests on the physical function of the hand and upper leg muscles were chosen to reflect everyday activities. The upper leg tests were adapted to avoid problems when performed by pregnant women, women who had recently given birth, and/or who had undergone a recent caesarean section.

#### Hand

Peak grip strength, defined as the highest value of three trials for each hand, was measured using a hand dynamometer (“GRIPPIT”, AB Detektor, Gothenburg, Sweden) and reported in Newtons (N) [35,36].

#### Upper leg

Four upper leg tests were performed and rated by the doctors as *done without effort* (able), or *not done* (unable):

a. Squatting. The person squatted and rose once;

b. Standing on one leg. Tested for 30 seconds [37];

c. Hip lifting test (Trendelenburg’s sign): the woman stood with one hand high up the wall and lifted the opposite leg for 30 seconds [19];

d. Ability to stand up from a chair (sit to stand): the person sat down and stood up five times with hands folded across their chest.

### Statistical analyses

Mean values with 95% confidence intervals (95% CI) were calculated for interval data, and median values (md) with inter-quartile ranges (IQR) calculated for ordinal data and small numbers.

The 25(OH)D concentrations were examined as: 1) a continuous variable normalized by transformation using the natural logarithm where the undetectable levels of 25(OH)D were replaced by the number ‘9’, and 2) categorized according to different standards, i.e. ‘Undetectable’ (<10 nmol/L), ‘Deficient’ (10–24 nmol/L), and ‘Insufficient’ (25–49 nmol/L); the higher levels were classified as ‘Adequate’ (50–75 nmol/L) and ‘Optimal’ (>75–250 nmol/L) [38].

Chi-square statistics, the Mann–Whitney U test, ANOVA, and t-tests were used to compare median and mean values between the two groups of women. Spearman’s rank correlation coefficient (rho) was calculated for grip strength across all 25(OH)D categories. Kruskal–Wallis and Mann–Whitney U tests were used to calculate significant differences in the distribution of the participants’ inability to perform the upper leg tests, across the 25(OH)D categories. Pearson correlation statistics were used to explore the uni-level association between 25(OH)D levels and grip strength.

A final main effect model was calculated using stepwise linear regression to examine significant predictors for maximal voluntary grip strength among the independent variables: country of birth, age, height, weight, 25(OH)D level, physical activity, gestational age, parity, and lactation status.

Two-sided significance tests were used. A p-value of 0.05 or less was considered statistically significant. Data were analyzed using IBM SPSS Statistics, version 17, New York, USA.

#### Ethical approval

The study was approved by the regional ethics committee located at Uppsala University (D 210/140), Sweden.

## Results

### General information

The study population included 123 women (71 Swedish; 52 Somali). The majority of both groups, Swedish (57%) and Somali (67%) reported that they were completely healthy, with the remainder reporting allergic or gastrointestinal problems. The groups of women differed significantly in a variety of ways. More Somali women were breastfeeding (72% vs. 29% of Swedish women [p < 0.05]), while 2% of Somali women also were breastfeeding when pregnant (versus nil in the Swedish group). Other significant differences between the Swedish and Somali groups included: age (mean 30.7 years; 95% CI 29.6–31.8, vs. 28.3 years; 95% CI 26.6–30.0, respectively, p < 0.05), education (mean 13.8 years; 95% CI 12.9–14, vs. 3.3 years; CI 2.3–4.4, respectively, p < 0.001), height (mean 167 cm; 95% CI 166–169, vs. 161 cm; 95% CI 160–163, respectively, p < 0.001), systolic blood pressure (mean 113 mmHg; 95% CI 110–115, vs. 104 mmHg; 95% CI 101–107, respectively, p < 0.001) and number of children (md 1; IQR 1–2, vs. 3; IQR 1–5, respectively, p < 0.001). Four Swedish and two Somali women had recently given birth, one of whom had undergone a cesarean section and therefore did not perform the squatting test. Four Somali women (4/52) had a hemoglobin count <100 g/L (89–99) during pregnancy. No woman had a history of significant blood loss during delivery. None of the women were receiving narcotics or anxiolytic analgesics; mild analgesics were the only on-going medications. The above variables did not vary significantly across the categories of 25(OH)D.

### 25(OH)D

Swedish women had higher reported 25(OH)D levels (p < 0.001; mean 49.5 nmol/L [41.1–53.8]) while the mean level for the Somali women could not be calculated because one third had undetectable values. Therefore, categories of 25(OH)D were used in the calculations instead (Table 1)*.* The first table shows that 90% of the Somali women had a 25(OH)D deficiency with levels <25 nmol/L. Notably, 35% of the Somali women had undetectable plasma 25(OH)D. The Somali women with low categories of 25(OH)D had particularly high PTH levels. Levels of PTH were not associated with gestational age, recent delivery, or lactation status.

**Table 1:** Distribution of the social and clinical data of 71 Swedish and 52 Somali women by categories of 25(OH)D (nmol/L), with mean values and 95% confidence interval (95% CI) of interval data and median values (IQR) of ordinal data, and number (n) and frequencies of categorical data

	**25(OH)D categories**					**p**
	**< 10**	**10–24**	**25–49**	**50–74**	**> 75**	
**Swedish, n (%)**		7 (9.9)	31 (43.7)	29 (40.8)	4 (5.6)	
**Somali, n (%)**	18 (34.6)	29 (55.8)	4 (7.7)	1 (1.9)		
**Variables**						
***PTH, mean 95% CI***						
**Swedish**		4.4 (2.5–6.4)	4.8 (3.8–5.7)	4.4 (3.4–5.3)	5.7 (0.6–10.8)	
**Somali**	16.2 (7.7–24.7)	11.1 (9.4-12.9)	6.8 (2.0–11.7)			
***Age, years***						
**Swedish**	–	31.7 (24.8–35.6)	29.9 (28.2–31.7)	31.2 (29.4–32.9)	31.0 (18.7–43.3)	
**Somali**	26.7 (23.4–30.0)	29.0 (26.7–31.4)	28.3 (16.7–35.8)	36.0		
***Height, cm***						
**Swedish**		164 (158–169)	168 (166–170)	167 (165–170)	171 (163–179)	
**Somali**	161 (159–163)	162 (159–164)	165 (160–169)	155		
***Pregnant, months***						
**Swedish**	–	6.3 (4.3–8.4)	6.1 (5.3–7.0)	5.9 (5.1–6.8)		
**Somali**	6.2 (5.4–7.0)	5.8 (3.7–8.0)	–			
***Parity, n***						
**Swedish**		1 (0–2)	1 (0–2)	1 (1–1.5)	1 (0.5–1)	**0.02**
**Somali**	1 (0–4)	3 (2–6)	2.5 (1–5.5)	3		
***Activity/week, n***						
**Swedish**	–	3 (0–3)	0 (0–3)	3 (0–3)	3 (1–3)	**0.04**
**Somali**	0 (0–3)	1.5 (0–3)	3 (0–3)			

Analysis of variance test of variance (ANOVA) for significant differences in means between groups of interval data. Kruskal-Wallis test was used for significant differences in medians across categories of 25(OH)D. PTH in pmol/L.

Significant p-values are in bold.

In comparison, 10% of Swedish women had a deficiency and 54% had insufficient levels, with levels <50 nmol/L. The mean value for the Swedish women was 50 nmol/L; only 6% of the Swedish women had levels >75 nmol/L.

Two variables differed significantly in distribution between the categories of 25(OH)D by country of birth. In the Somali group, the number of children increased in the higher 25(OH)D category (p = 0.024). In the Swedish group, the number of weekly physical activities increased in the higher 25(OH)D categories (p = 0.04); they also performed significantly better in three of the four upper leg tests (p < 0.001); the sit to stand test was the exception (see Table 2).

**Table 2:** Categories of 25(OH)D concentrations (nmol/L) and the median (md) values of grip strength (0–600 Newtons) with inter-quartile ranges (IQR) and the number (%) of women by country of birth who were unable to perform the upper leg muscle tests unaided

**25(OH)D categories**	**< 10**	**10–24**	**25–49**	**50–74**	**≥ 75**	
Swedish, n		8	31	28	4	
Somali, n	18	29	4	1		
						Rho^a^ p
*Grip strength,* md (IQR)	173 (143–219)	223 (189–258)	311 (267–354)	326 (294–370)	313 (303–415)	**0.68 <.001**
Swedish		264* (202–327)	318* (293–344)	327 (305–349)	313 (303–415)	0.21 .078
Somali	173 (143–219)	211 (187–244)	223 (106–285)	292		**0.35 .012**
Inability to: n (%)						p^b^
*Squat*						
Swedish		0	0	0	0	
Somali	15 (83.3)	18 (64.3)	4 (100)	1		.33
*Sit to Stand*						
Swedish		0	0	0	0	
Somali	1 (5.6)	1 (3.6)	0	0		
*Trendelenburg*						
Swedish	0	1	0	0	0	.65
Somali	5 (27.8)	5 (17.2)	1 (25.0)	0		.46
*Stand on one leg*						
Swedish			2 (6.5)	1 (3.6)	1 (25.0)	.57
Somali	5 (27.8)	8 (27.6)	1 (25.5)	1 (100)	–	.72

^a^Spearman rho with p-values below.

*p < 0.05 Mann–Whitney U test compared with the group below.

^b^Tests of distribution across 25(OH)D categories by Kruskal-Wallis test.

Significant p-values are in bold.

Table 2 shows that the Swedish women had a significantly stronger median grip strength than the Somalis in the ‘10–24’ and ‘25–49’ 25(OH)D categories (264 N, Swedish vs. 211 N, Somali and 318 N, Swedish vs. 223 N, Somali, for the respective plasma ranges, both p < 0.05). It also shows that grip strength correlated with 25(OH)D categories: overall Spearman rho 0.68; p < 0.001; by country: Somali rho 0.35 (p = 0.01), Swedish rho 0.21 (p = 0.078). Three quarters (73%) of the Somali women were unable to squat, and one fifth (21%) had a positive Trendelenburg’s sign, but there were no statistically significant differences in distribution across the 25(OH)D categories. In addition, 29% of the Somali women were unable to stand on one leg; these results did not reach statistical significance across all of the 25(OH)D categories. In addition, there were no significant differences in test results between lactating and non-lactating women (chi-square test).

**Figure 1:**
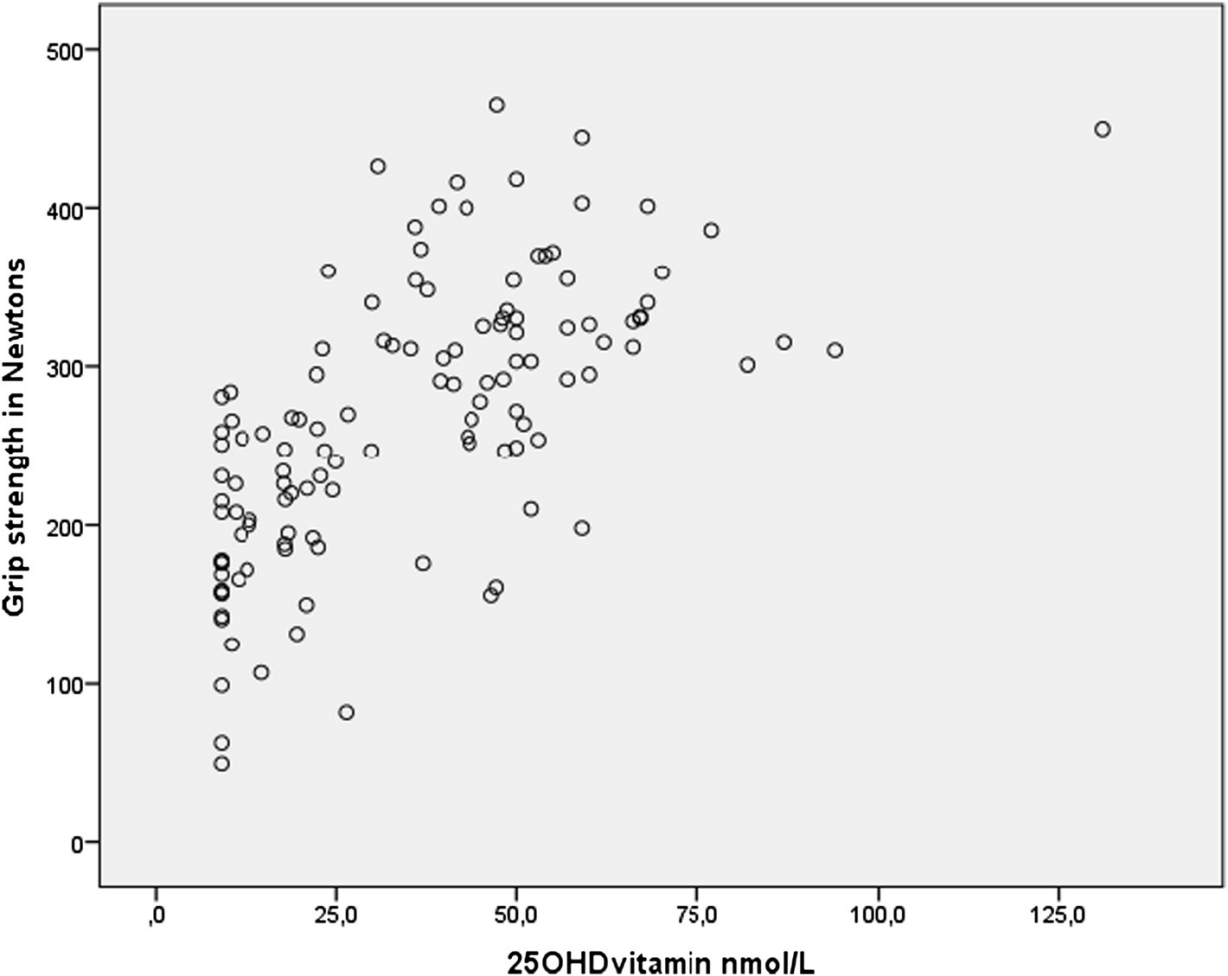
**Pearson correlation coefficient corr. 0.65, p < 0.001.** Undetectable values of 25(OH)D were replaced with the number ‘9’ in the calculation.

Figure 1 illustrates that the 25(OH)D concentrations (nmol/L) and grip strength (N) had a significant linear correlation (0.65, p < 0.001).

The final main effect model in Table 3 shows that grip strength (N) was predicted by the 25(OH)D levels (B 0.94, p = 0.013), adjusted for country of birth (B −63.9, p < 0.001), age (B 2.5, p = 0.015) and height (B 2.6, p = 0.012). Physical activity, lactation status, parity and gestational age had no significant relationship with observed grip strength.

**Table 3:** Final main effect model using linear regression with stepwise exclusion for maximal voluntary grip strength in 123 pregnant women and new mothers

**Variable**	**B**	**95% CI**	**p**
25(OH)D	0.94	(0.21–1.68)	**.013**
Country	63.9	(-97.9 – -29.9)	**<.001**
Age	2.5	(0.15 – 4.60)	**.015**
Height	2.6	(0.59 – 4.65)	**.012**

Independent variables included at the first step: country of birth, 25(OH)D level, age, height, weight, physical activity level, lactation status, parity, gestational age.

Unstandardized (B) with 95% confidence intervals (95% CI) and p-values. Significant p-values are in bold.

## Discussion

In summary, one third of the Somali women had undetectable (<10 nmol/L) plasma 25(OH)D, whereas nearly half the Swedish women had levels >50 nmol/L. Furthermore, nearly all Swedish women had a much stronger grip than the average Somali woman. Grip strength was linearly associated with plasma 25(OH)D, age and height, and also with country of birth. In addition, most Somali women had obvious problems with weak upper leg muscles, but this finding had no statistically significant association with low 25(OH)D levels.

Our hypothesis was partly confirmed because only grip strength had a statistically significantly association with the 25(OH)D concentrations. Other factors such as lifestyle and socio-cultural attitudes to gender roles, age, and physical activity levels may have contributed to the poor physical performance among our Somali women, especially regarding their upper leg function; this finding has been reported by other authors [39]. Further studies are warranted to bridge this knowledge gap.

As in other studies, we observed that the concentrations of 25(OH)D did not differ significantly between the pregnant women and new mothers [40]. Many pregnant immigrant women seem to have low 25(OH)D concentrations [41]. In the United Kingdom, it was found that more than half of recent mothers with immigrant backgrounds had 25(OH)D <25 nmol/L, and another 15% had <12.5 nmol/L [4,42]. Of note, the Somali women in our study had much lower levels than in that study, and a sizeable proportion had undetectable concentrations.

In total, 93% of our Swedish women had a grip-strength above the Somali median value of 202 N, a value close to that performed by Swedish women in another study [35]. However, the mean grip strength of our Somali women was comparable to values measured in pregnant Ethiopian women [43]. Overall, the poor physical performance observed in the current study implies poor general health in this sub-group of pregnant women. This impairment could affect their ability to nurse their newborn babies [44]. Furthermore, low plasma 25(OH)D might also cause poor intrauterine contractions during labor [25,27].

The association between 25(OH)D concentrations and muscular performance is well-known, particularly in elderly persons [20]. In a Danish study of 55 veiled Arabic women and 22 Danish controls, vitamin D treatment improved quadriceps muscle power [7,9]. Ultraviolet B radiation has also been suggested to improve athletic performance by elevating plasma 25(OH)D [2]. Our study contributes further evidence, in that even after long-standing deprivation of ultraviolet B radiation, the 25(OH)D concentration correlates to hand-grip strength, which confirms that ultraviolet B radiation affects muscle strength via 25(OH)D [2]. Recently, vitamin D requirements in the United States were revised upwards with regard to soft-tissue health [45]. There is currently no consensus in Sweden for cut-off points for vitamin D insufficiency based on serum 25(OH)D [40].

The independent variable in this study was 25(OH)D. It was crucial that the method used to measure it was valid and reliable. There are different methods for measuring vitamin D and its free variants [46]. Our laboratory had consistently lower values by 10–20% than the specific LC-MS reference methods used at other laboratories [24,47].

Notably, PTH affects calcium levels via the “classical pathway” compared with the proportional effect of 25(OH)D that was the focus here. Many of our Somali women had elevated, and even high PTH plasma concentrations. PTH is measured rarely in primary care practice. In this instance, PTH was measured only to indicate resorption of bone mass. In comparison, in another study researchers examined the combined effects of vitamin D and PTH on physical performance [12].

### Strengths and limitations

This is the first Swedish study that has demonstrated extremely low concentrations of vitamin D in Swedish residents who have immigrated from Somalia. This is the first Swedish study about the correlation between physical performance and 25(OH)D plasma measurements. To the best of our knowledge, there are no previous published studies on grip strength, upper leg performance, and 25(OH)D levels in pregnant, newly pregnant, lactating darkly-pigmented veiled women, or Swedish pregnant women living in the high northern latitudes. In addition, this study is one of first that concerns vitamin D status in pregnant women in Sweden; it has shown that 25(OH)D deficiency also exists in younger Swedish women during spring, and confirms that Somali–Swedish women are often severely deficient in vitamin D, with important consequences for many target organs. The strength of our research lies in its real-world cross-sectional design, with blood and physical tests performed during a short defined period after winter. However, the results presented here must be interpreted with caution and should not be widely generalized, but it may be possible to transfer the findings to similar populations. A limitation is the rather small study group. All of the findings from this study are preliminary and need further exploratory studies, e.g. relating to: attitudes to sun exposure, levels of sun exposure, veiling, food intake, breastfeeding during pregnancy, and the role and implications of darkly-pigmented skin in real life.

### Clinical significance

Maternity health care personnel should be aware of the increased frequency of severe 25(OH)D deficiency among young women exposed to diminutive sun radiation because of dark skin pigmentation and the use of protective clothing. Notably, a grip power below 20 kg (200 N) is likely to have negative effects on childcare and housework, and may also increase the risk of accidents. Health equity in antenatal care requires focus on nutrition status, especially in migrant women [48].

## Conclusions

Many of the Somali women in this study had undetectable or very low 25(OH)D concentrations and pronounced muscle weakness in the hands and upper legs, where grip strength was strongly associated with low 25(OH)D concentrations. These findings have implications for the antenatal care of this population. Maternity health care personnel should be aware of this increased frequency and manage their care accordingly.

## Competing interests

The authors declare that they have no competing or conflicts of interests with respect to this research.

## Authors’ contributions

PK conceived the study design, conducted the study, performed the analysis, interpretation of data and drafted the manuscript. YB participated in the design of the study and helped to draft the manuscript. ML designed the study, helped to perform the analysis, interpreted the data, and drafted the manuscript. All authors read and approved the final manuscript prior to submission.

## Pre-publication history

The pre-publication history for this paper can be accessed here:

http://www.biomedcentral.com/1471-2393/13/237/prepub
